# New insights into tenocyte-immune cell interplay in an *in vitro* model of inflammation

**DOI:** 10.1038/s41598-017-09875-x

**Published:** 2017-08-29

**Authors:** Meaghan Stolk, Franka Klatte-Schulz, Aysha Schmock, Susann Minkwitz, Britt Wildemann, Martina Seifert

**Affiliations:** 1 Berlin-Brandenburg Center for Regenerative Therapies (BCRT), Charité-Universitätsmedizin Berlin, corporate member of Freie Universität Berlin, Humboldt-Universität zu Berlin, and Berlin Institute of Health, Berlin, 13353 Germany; 2 Julius Wolff Institute, Charité-Universitätsmedizin Berlin, corporate member of Freie Universität Berlin, Humboldt-Universität zu Berlin, and Berlin Institute of Health, Berlin, 13353 Germany; 3 Institute of Medical Immunology, Charité-Universitätsmedizin Berlin, corporate member of Freie Universität Berlin, Humboldt-Universität zu Berlin, and Berlin Institute of Health, Berlin, 13353 Germany

## Abstract

Inflammation plays an important role in the development and resolution of tendon diseases, but underlying mechanisms are poorly understood. We therefore aimed to analyze the response of human tenocytes to inflammatory stimuli and to uncover their interplay with macrophages *in vitro*. Tenocytes from human ruptured supraspinatus tendons (n = 10) were treated for three days with a stimulation mixture derived from activated mononuclear cells isolated from healthy human peripheral blood. Significantly increased expression levels of selected adhesion- and human leukocyte antigen (HLA)-molecules, and enhanced interleukin (IL)-6 release were detected by flow cytometry. Tenocyte stimulation with the pro-inflammatory cytokines interferon gamma, tumor necrosis factor alpha and IL-1ß triggered similar changes in surface markers and enhanced the release of IL-6, IL-8 and monocyte chemoattractant protein 1 (MCP-1). In co-cultures of macrophages with pre-stimulated tenocytes, macrophages significantly increased CD80 expression, but simultaneously decreased HLA-DR-expression, which are both typical pro-inflammatory polarization markers. Co-cultures also released more IL-6, IL-8, MCP-1 than tenocyte-cultures alone. We demonstrate that tenocytes respond to inflammatory environments *in vitro* with altered surface marker and cytokine profiles and influence macrophage polarization. Importantly, all changes detected in direct co-cultures were also present in a transwell setting, implicating that communication between the cells involves soluble factors.

## Introduction

Tendon ruptures can result from either acute traumatic injury or follow chronic overuse leading to degeneration. Healing after tendon reconstruction remains a problem of high clinical relevance, due to the often unsatisfactory patient outcomes with high rates of recurrent defects or non-healing^1, 2^. An acute tendon injury can thereby turn into a chronic pathology, whereas the biological reason for this incomplete healing is still unclear.

Recently, the role of inflammation in the development of chronic degenerative tendon pathologies (tendinopathies) has been explored^3^. Understanding has been improved using evidence accumulated by analyzing patient samples from different disease stages^4–6^. Immunohistochemical studies clearly confirmed that inflammatory cells such as mast cells, T cells and macrophages are present in early human tendinopathy^7, 8^. The impact of direct cytokine stimulation on tenocytes has also been investigated, but studies were limited to using mixed tenocyte sources and examining changes mainly at the gene level^5, 9^.

Inflammation in injured tendons is characterized by the infiltration of immune cells such as neutrophils and macrophages. Initially, pro-inflammatory macrophages release cytokines into the repair site and promote tendon extracellular matrix (ECM) degradation, inflammation and apoptosis. In the later stages of acute tendon healing, tissue repair macrophages dominate, and release anti-inflammatory cytokines to dampen inflammation and promote tendon remodeling^10–12^. Therefore, the balance between pro- and anti-inflammatory cells and soluble factors within the tendon healing process will have an important impact on the successful resolution of inflammation. Recent analyses of ruptured supraspinatus tendon samples have shown a distinct inflammatory infiltrate within the early phase of the disease containing elevated pro-inflammatory cytokines such as IL-6, TNFα and IL-17^13^. Pro-inflammatory factor release in an early phase of tendinopathy could also impact collagen production in general, or the type of collagen produced, as demonstrated by IL-33 induced inhibition of miR-29a^14^.

Although the presence of macrophages in injured tendons is now known, their exact impact on tenocytes themselves and tendon repair is unclear. The role of monocyte derived macrophages for tissue remodeling and repair is known from many other tissues and diseases^15–18^, and is likely important in tendon injury as infiltration of CD14^+^CD68^+^ myeloid cells was already demonstrated, mostly by immunohistochemistry, flow cytometry or mRNA expression analyses^7, 13, 19, 20^.

Tissue macrophages are known to become activated and polarized under the influence of the surrounding matrix and factor environment^21^. Key macrophage polarization markers in terms of phenotype, gene expression or signaling pathways have emerged from experimental data to identify broadly pro-inflammatory M1 and anti-inflammatory M2 subsets, and their further subdivision into M2a-c subtypes^15, 22^. However, macrophage polarization rather represents a continuum with the environment giving cues for polarization direction rather than the development of a fixed cell type^23, 24^. Hence polarization is more of a reversible and dynamic process than initially thought. The balance of M1/M2 type polarization clearly influences the final repair outcome, and inadequate or dysregulated resolution of the inflammation would lead to chronic inflammation and fibrosis^25^. Specific inflammation signaling during different phases of tendinopathy and affecting macrophage polarization was recently shown by analyzing patient samples^5^. Although considerable progress has been made in understanding the inflammatory processes within the acute phase of human tendinopathies, the interplay of tendon resident mesenchymal cells with immune cells, as well as the surrounding matrix and soluble factor environment is still poorly understood.

Therefore, the aim of the present study is to analyze the *in vitro* responses of human tenocytes from ruptured supraspinatus tendons to pro-inflammatory factors. We hypothesize that soluble factors originating from activated immune cells or the direct contact and interaction of tenocytes with macrophages will affect tenocyte surface marker expression, the amount of soluble factors released, and alter the polarization state of the macrophages.

## Results

### Responses of tenocytes to a pro-inflammatory immune cell derived stimulus

**Figure 1:**
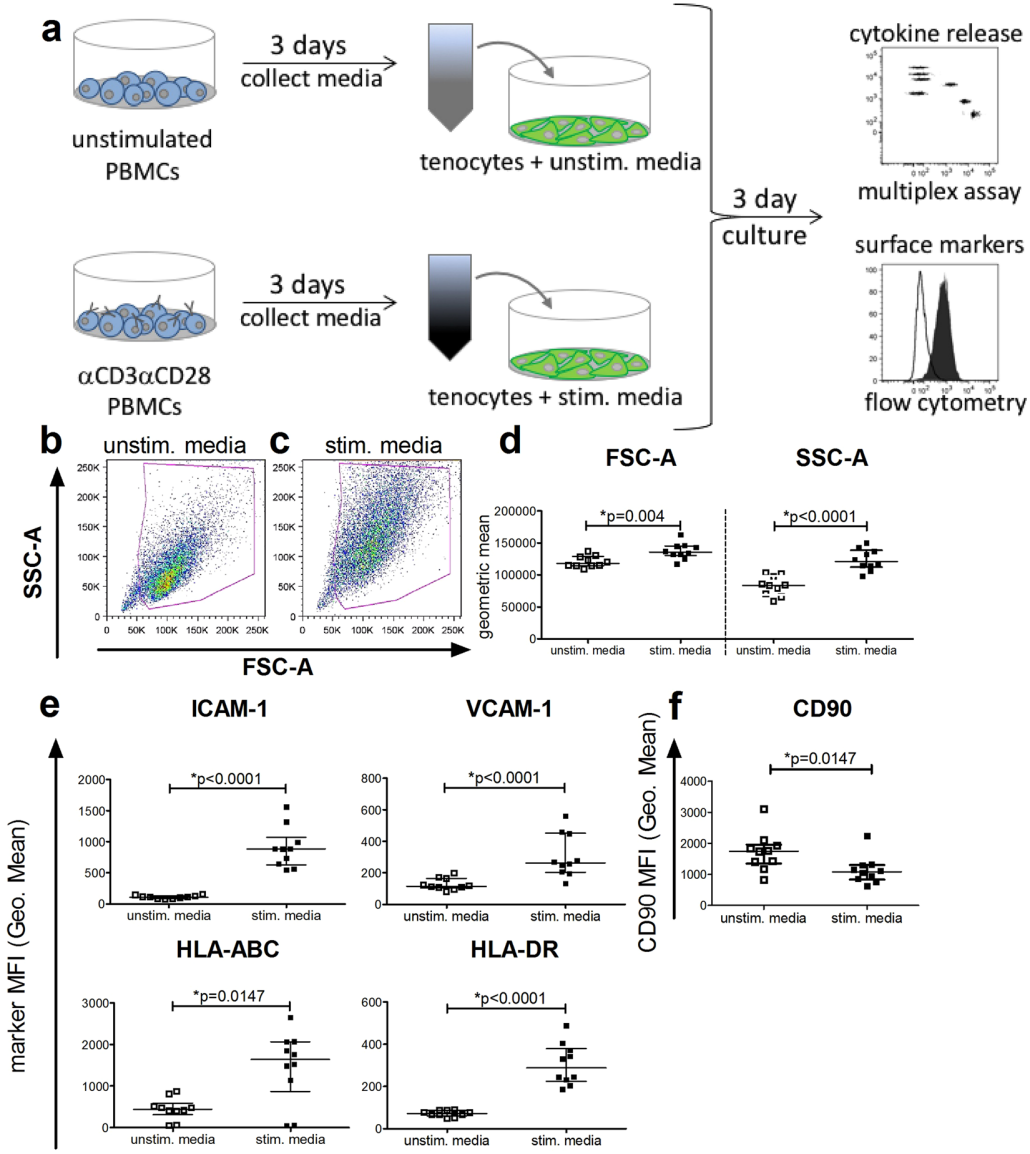
Pro-inflammatory stimulation media increases the size and granularity of tenocytes, and changes their surface marker expression. Following incubation for 3 days with either the control media from unstimulated PBMC (unstim. media) or the pro-inflammatory stimulation media from αCD3αCD28 stimulated PBMCs (stim. media) (**a**), supernatants were collected for later cytokine evaluation before the tenocytes were stained with a panel of human-specific antibodies to characteristic surface markers and analyzed by flow cytometry. Tenocytes included in the viable gate with unstim. media (**b**) or with the stim. media (**c**) are shown here with representative flow cytometry density plots. The mean fluorescence intensity (MFI) of forward scatter area (FSC-A) and sidewards scatter area (SSC-A) indicating cell size and granularity are shown (**d**). The expression of the adhesion molecules ICAM-1 (CD54) and VCAM-1 (CD106), as well as the HLA class I molecule HLA-ABC and the HLA class II molecule HLA-DR (**e**) and CD90 (Thy-1) (**f**) are also shown after incubation with unstimulated and stimulated media. Data are presented as the median of n = 10 with the interquartile range and are considered significantly different when *p < 0.05 with the Mann-Whitney non-parametric t-test.

To mimic the clinically relevant inflammatory conditions that might influence tenocytes during tissue injury, a complex stimulation media from αCD3αCD28 stimulated PBMC cultures containing a set of human cytokines, predominantly IL-6, IL-8, MCP-1, TNFα, and IFNγ was generated (Supplemental Fig. 1). Tenocytes were subjected to either the control unstimulated media or the stimulation media derived from αCD3αCD28 stimulated PBMCs (Fig. 1a) for 3 days, and then stained with a multicolor panel of anti-human specific antibodies against surface antigens for flow cytometry analysis. Compared to the control unstimulated media (Fig. 1b), culturing with the stimulation media (Fig. 1c) increased the geometric mean of both the forward scatter (FSC-A) and the sidewards scatter (SSC-A), resulting in significantly increased size and granularity (Fig. 1d). Stimulation also led to a significant increase in the expression of the adhesion molecules ICAM-1 (CD54) and VCAM-1 (CD106). HLA molecules are important markers for immune recognition and antigen presentation and were significantly up-regulated on the tenocytes after stimulation with an increase of HLA-ABC (HLA class I) and HLA-DR (HLA class II) (Fig. 1e). In contrast, CD90 (Thy-1) showed significantly reduced expression after stimulation (Fig. 1f). Other surface markers found on tenocytes such as CD105, CD29, CD44 and CD73 were not affected by stimulation (Supplemental Fig. 2).

**Figure 2:**
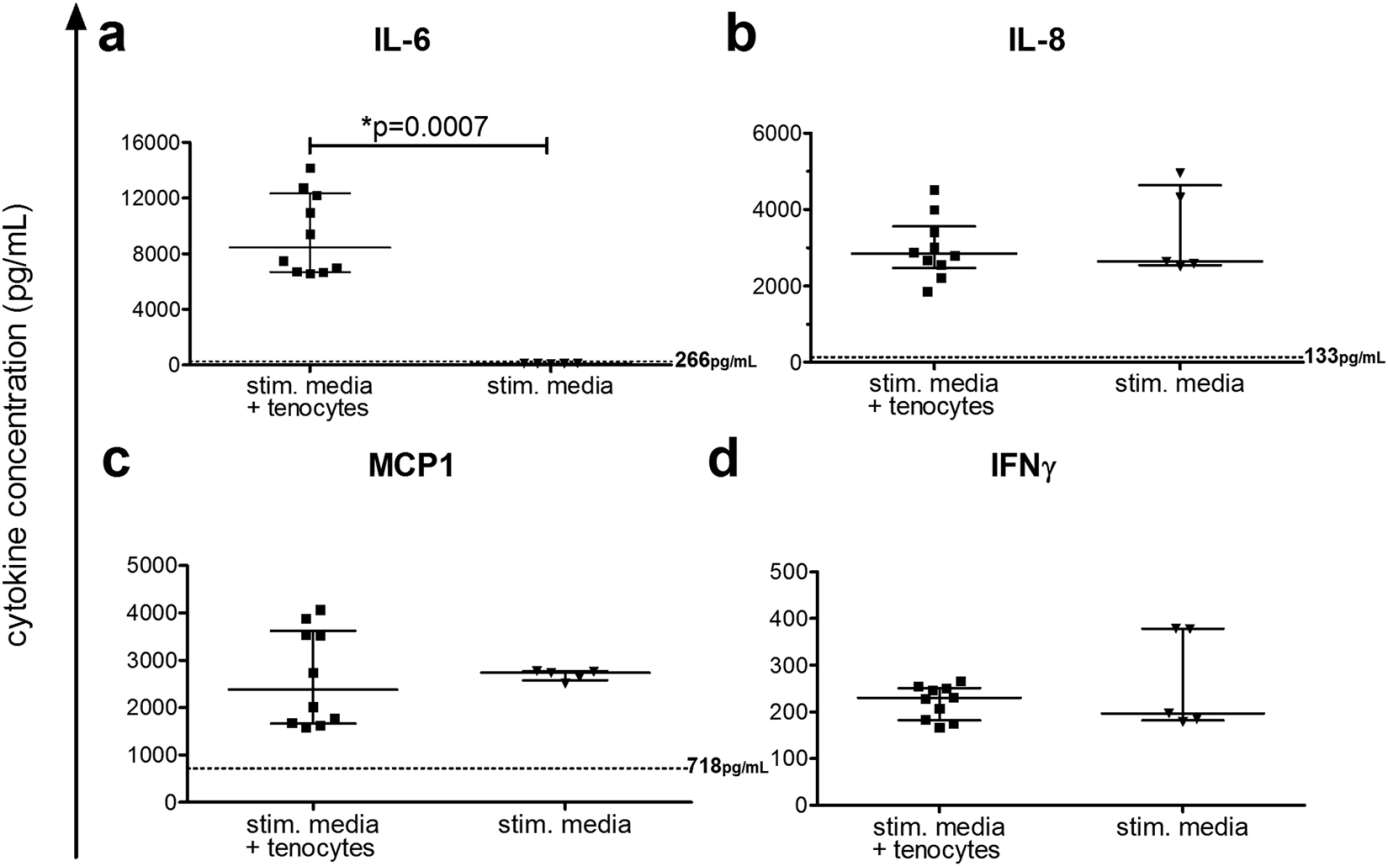
Tenocytes produce IL-6 upon stimulation. Cytokines were measured in media from αCD3αCD28 stimulated PBMCs (stim. media) (see Fig. 1a) using the Legendplex^TM^ human inflammation panel. After tenocytes were cultured for 3 days with either the control media from unstimulated PBMC (unstim. media + tenocytes; dotted line) or stimulation media (stim. media + tenocytes), cytokines were measured in the supernatants. The levels of IL-6 (**a**), IL-8 (**b**), MCP-1 (**c**) and IFNγ (**d**) are shown for the tenocytes cultured with stimulated media compared to the stimulated media without tenocytes present. The median levels for tenocytes incubated with the unstimulated media are shown next to the dotted line at that level on **a–c**, and IFNγ was undetectable. Samples were treated following the manufacturer’s instructions and measured by flow cytometry. Analysis was done using Legendplex software. Data are presented as the median with the interquartile range with an n = 5 for stimulated media alone and n = 10 for stimulated media + tenocytes, and are considered significantly different when *p < 0.05 with the Mann-Whitney non-parametric t-test.

After incubation of the tenocytes for 3 days with the medium from the stimulated PBMC, the cytokine levels were evaluated. Tenocyte cultures subjected to the control unstimulated media released basal levels of IL-6 (median 266 [interquartile range 155–445] pg/mL), IL-8 (133 [125–151] pg/mL), and MCP-1 (718 [636–932] pg/mL) and no IFNγ. The amount of IL-6 present in the supernatant significantly increased for tenocytes cultured with the stimulation media compared to the IL-6 level of the stimulation media itself (Fig. 2a). No further release of the factors IL-8, MCP-1 and IFNγ was measured after culture with tenocytes (Fig. 2b–d).

### Tenocyte reponses to essential pro-inflammatory cytokines

**Figure 3:**
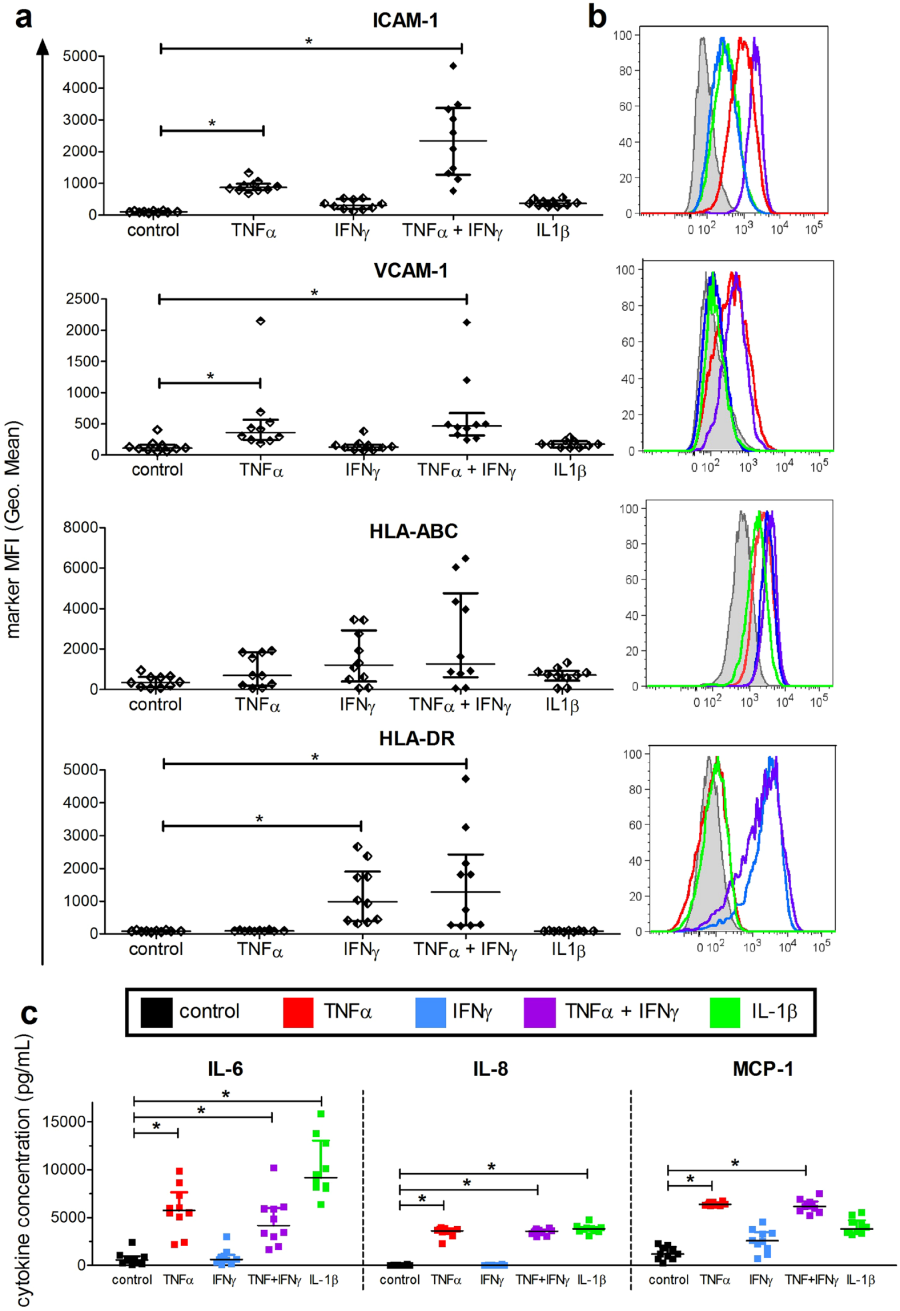
Increased marker expression and cytokine release after stimulation of tenocytes with single or combined pro-inflammatory recombinant cytokines. Tenocytes were seeded overnight and then recombinant cytokines were added for a final concentration of 10 ng/mL. Following incubation for 3 days, supernatants were collected before tenocytes were evaluated by flow cytometry for various markers. The mean fluorescence intensity (MFI) of ICAM-1 (CD54), VCAM-1 (CD106), HLA-ABC and HLA-DR was evaluated (**a**). Representative histogram overlays for each marker following stimulation with TNFα (red), IFNγ (blue), TNFα + IFNγ (purple) and IL-1ß (green) compared to the unstimulated control (black) are shown in (**b**). Supernatants from the cultures were evaluated with the Legendplex^TM^ human inflammation panel. Shown are the summarized data for IL-6, IL-8 and MCP-1 levels in tenocyte cultures after stimulation with the cytokines in comparsion to the unstimulated control (**c**). All data are presented as the median of n = 10 with the interquartile range and are considered significantly different when *p < 0.05 with the Kruskal Wallis non-parametric ANOVA with Dunn’s post-test.

Selected cytokines (TNFα, IFNγ, and IL-1ß) that are mainly released by activated and tissue invading monocytes were analyzed with regard to their effects on tenocyte features. Initial experiments showed that stimulation of tenocytes with 10, 25, or 50 ng/mL showed similar increases in the surface markers ICAM-1, VCAM-1 and HLA-ABC, and that stimulation for 3 days was required for upregulation of HLA-DR (data not shown), so the timepoint of 3 days with 10 ng/mL cytokine stimulation was chosen. Following the stimulation, tenocytes were harvested and evaluated with flow cytometry for the mean fluorescence intensity (MFI) of various markers. The amount of ICAM-1 (CD54) increased significantly with TNFα (Fig. 3a) and rose further with the combination of TNFα and IFNγ. Similarly, VCAM-1 (CD106) increased with TNFα by itself and when in combination with IFNγ compared to unstimulated tenocytes. Moreover, the amount of HLA-DR on the tenocytes increased significantly with IFNγ alone or in combination with TNFα, whereas the expression level of HLA-ABC only showed a trend of increasing upon cytokine stimulation. CD90 was not affected (data not shown). Representative histogram overlays for ICAM-1 and HLA-DR expression under different cytokine stimulation are displayed in Fig. 3b. Other cytokines found in the stimulation media (IL-6, IL-8 and MCP-1) were also tested alone or in combination at 10 ng/mL on the tenocytes, but no significant differences regarding marker expression were measured compared to the control unstimulated media (Supplemental Fig. 3).

Additionally, the supernatants from the cytokine-stimulated tenocytes were evaluated for the presence of 13 cytokines. In Fig. 3c, the summarized data for IL-6, IL-8 and MCP-1 are shown with significant increases detected upon stimulation with TNFα by itself or in combination with IFNγ compared to unstimulated tenocytes. The amount of IL-6 and IL-8 also increased significantly with IL-1ß stimulation. Accumulation of MCP-1 in the supernatant appeared elevated with IFNγ and IL-1ß, though these increases were not significant. Other cytokines measured including IL-1β, IFNα, IFNγ, TNFα, IL-10, IL-12p70, IL-17A, IL-18, IL-23, and IL-33 were detected at levels below 10 pg/mL except for when the stimulating cytokines TNFα, IFNγ and IL-1ß had been added (data not shown).

### Communication between human macrophages and tenocytes in co-cultures

**Figure 4:**
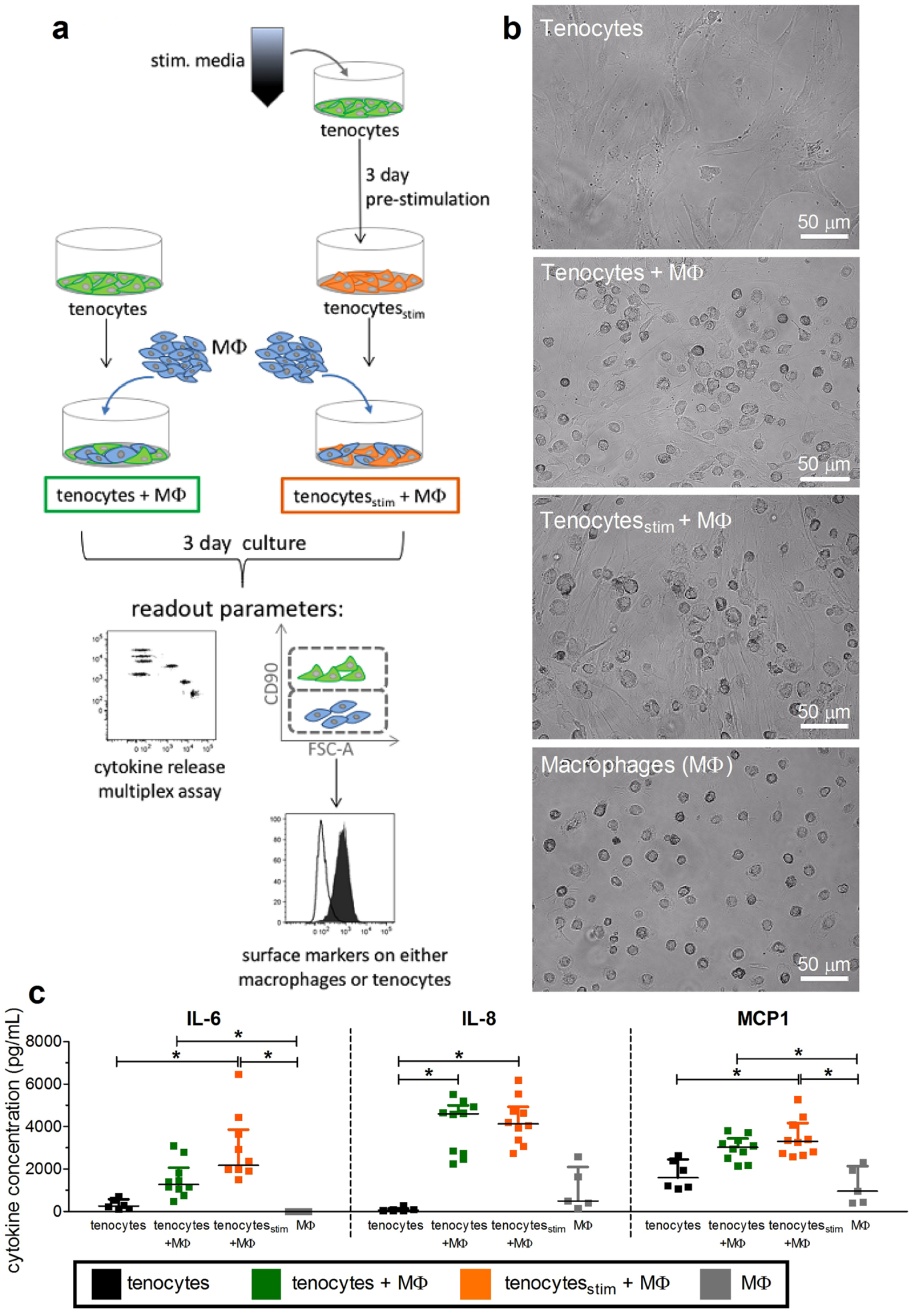
Co-cultures of tenocytes with macrophages increases the release of cytokines. A schematic representation of tenocyte and macrophage direct co-cultures is presented in (**a**). Tenocytes were either pre-stimulated with a mix of 50% media and 50% stimulation media for 3 days (tenocytes_stim_), or directly seeded into a 6 well plate. One day later, M0 macrophages (MΦ) derived from CD14^+^ monocytes using 50 ng/mL M-CSF for 6 days were added in a ratio of 1 tenocyte:5 macrophages to obtain co-cultures (tenocytes + MΦ) and pre-stimulated co-cultures (tenocytes_stim_ + MΦ). Additional wells of MΦ or tenocytes alone were seeded as controls. After 3 days of culture, supernatants were collected for later cytokine analysis before photos were taken of the cell cultures (**b**). Supernatants were evaluated using the Legendplex^TM^ human inflammation panel and the levels of IL-6, IL-8, and MCP-1 are shown (**c**). Data are presented as the median with the interquartile range of n = 5–6 for either tenocytes or macrophages alone and n = 10 for tenocyte/macrophage co-cultures and are considered significantly different when *p < 0.05 with the Kruskal Wallis non-parametric ANOVA with Dunn’s post-test.

Monocyte derived macrophages are the main source of diverse pro-inflammatory cytokines and chemokines, and are one of the first cell subsets that are in contact with tenocytes during tendon injury. Therefore, the interplay of tenocytes with macrophages was analyzed using co-cultures. The general experimental setup is shown in Fig. 4a. M0-type macrophages were generated by a 6 day M-CSF stimulation of blood-derived CD14^+^ monocytes, and added to tenocytes which were seeded at day-1, with or without pre-stimulation for 3 days using the stimulation media derived from αCD3αCD28 stimulated PBMCs. Tenocytes and macrophages were co-cultured in a ratio of 1 to 5, and after the 3 day co-culture, cell morphology, cytokine release, and surface marker expression were analyzed. Both cell types can be clearly distinguished by their morphology, and macrophages are more irregularly shaped after direct co-culture than when they were cultured alone (Fig. 4b). Additionally, the supernatants from the tenocytes alone or in direct co-culture with macrophages were evaluated for the presence of cytokines. A significant increase in IL-8 occurred when macrophages were co-cultured with tenocytes (Fig. 4c). The amount of IL-6, IL-8 and MCP-1 increased significantly when tenocytes were pre-stimulated before they were co-cultured directly with macrophages. Macrophages alone made no IL-6 and moderate values of IL-8 and MCP-1, while co-cultures with tenocytes produced significantly more IL-6 and MCP-1. Other cytokines measured including IL-1β, IFNα, IFNγ, TNFα, IL-10, IL-12p70, IL-17A, IL-18, IL-23, and IL-33 were detected at levels below 10 pg/mL (data not shown).

**Figure 5:**
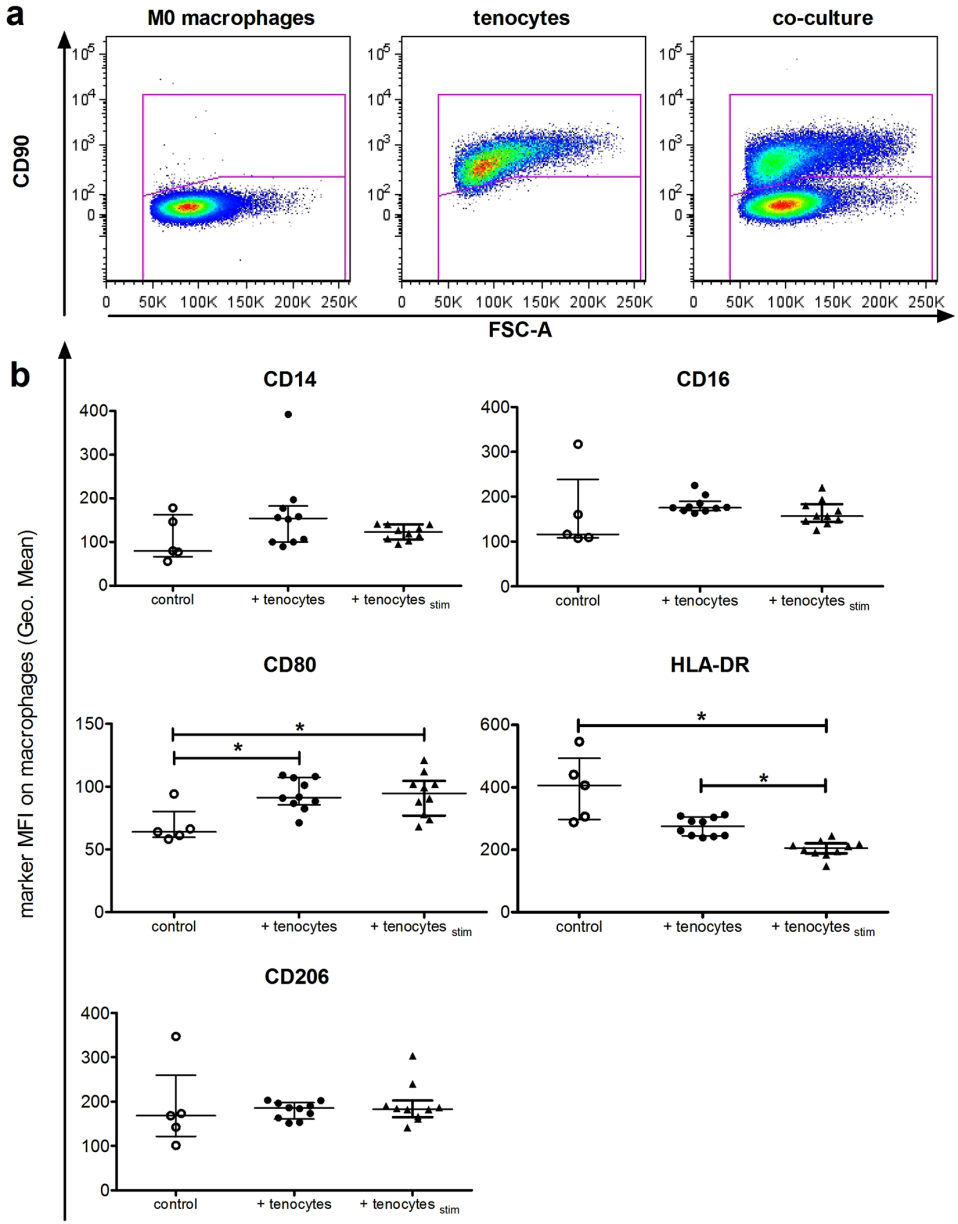
Co-culture with tenocytes changes the surface marker expression on macrophages. M0 macrophages were co-cultured together with tenocytes (+ tenocytes) or pre-stimulated tenocytes (+ tenocytes_stim_) in 6 well plates for 3 days. Additional wells of M0 macrophages alone (control) were cultured under the same conditions. After supernatants and culture photographs were taken, the cells were stained with a panel of human-specific antibodies to macrophage surface markers. Flow cytometry analysis of markers on the macrophages was achieved by first gating on the CD90 negative population to identify macrophages from the direct co-cultures (**a**). Shown is the mean fluorescence intensity (MFI) of CD14, CD16, CD80, HLA-DR and CD206 on macrophages from co-cultures with unstimulated or pre-stimulated tenocytes in comparison to control M0 macrophages (**b**). Data are presented as the median with interquartile range for an n = 5 for macrophages alone or n = 10 for tenocyte/macrophage co-cultures and are considered significantly different when *p < 0.05 with the Kruskal Wallis non-parametric ANOVA with Dunn’s post-test.

**Figure 6:**
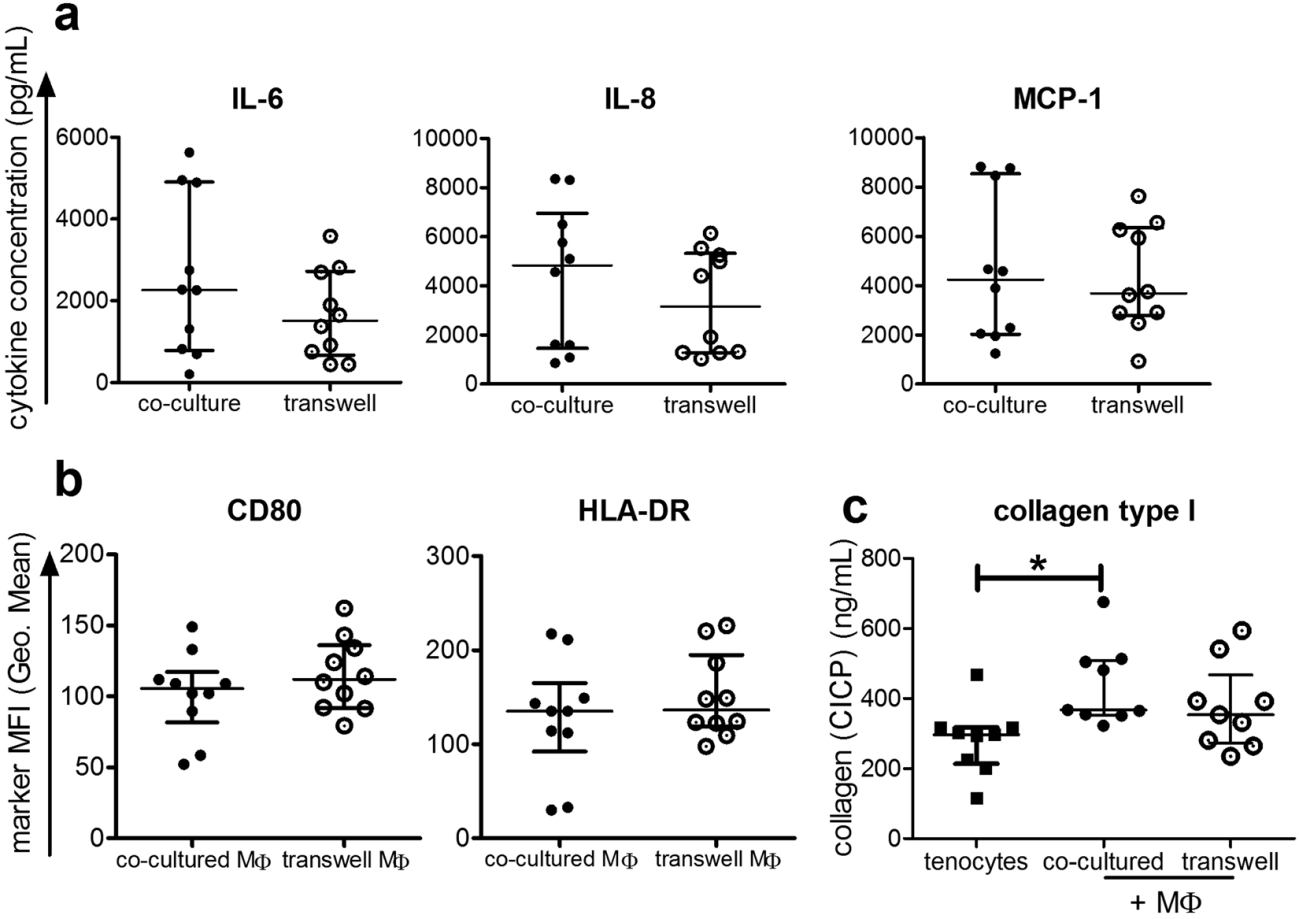
Soluble factors released in co-cultures were similar when performed in transwell assays. Supernatants were evaluated using the Legendplex^TM^ human inflammation panel and the levels of IL-6, IL-8, and MCP-1 are shown for either direct co-culture or transwell assays where tenocytes were cultured in inserts above adherent macrophages (**a**). The mean fluorescence intensity (MFI) is shown for CD80 and HLA-DR on macrophages following direct co culture (co-cultured MΦ) or transwell co-culture (transwell MΦ) with tenocytes (**b**). Supernatants were tested for CICP (C-terminal of type I collagen) using an EIA Kit from tenocytes alone and tenocytes co-cultured with macrophages directly or in transwell assays (**c**). Data are presented as the median with the interquartile range of n = 10 for (**a**) and (**b**) and n = 9 for (**c**), and are considered significantly different when *p < 0.05 with the Kruskal Wallis non-parametric ANOVA with Dunn’s post-test.

After initially gating on all single and viable cells (Supplemental Fig. 4), a gating strategy based on CD90 was used to discriminate between the surface marker expression on macrophages and tenocytes that had been directly co-cultured, as CD90 is expressed on tenocytes, but not on macrophages (Fig. 5a). Then the mean fluorescence intensity (MFI) for common monocyte/macrophage markers (CD14, CD16) and characteristic macrophage polarization markers CD80, HLA-DR (M1-type macrophages) and CD206 (M2-type macrophages) were determined on the CD90 negative cells. Significant increases in CD80 and a trend towards more CD14, CD16 and CD206 occurred upon direct co-culture with tenocytes compared to M0 type macrophages alone (Fig. 5b). Surprisingly, the expression level of HLA-DR appeared to decrease on macrophages when tenocytes were present, and decreased further to become significantly reduced when the tenocytes had been pre-stimulated with the stimulation media mixture before their initial seeding, designated as pre-stimulated tenocytes (tenocytes_stim_). Pre-stimulation did not affect the other macrophage surface markers. After co-culture with macrophages, the surface markers ICAM-1 (CD54) and VCAM-1 (CD106) on tenocytes were elevated, but HLA-ABC and HLA-DR were not affected (Supplemental Fig. 5). To determine whether effects were contact dependent in the direct co-cultures, some assays were repeated using a transwell system. The amounts of IL-6, IL-8, and MCP-1 measured were not significantly different in the transwell system compared to direct co-cultures (Fig. 6a). Macrophages co-cultured with tenocytes, but without direct contact in the transwell system, had unchanged levels of the surface markers CD80 and HLA-DR as compared to the direct co-culture (Fig. 6b). Co-culture of tenocytes with macrophages elevated their collagen production as CICP (C-Terminal of Type I Collagen) values in supernatants were higher compared to tenocytes alone, though only direct co-cultures showed a significant increase (Fig. 6c). Collagen production by tenocytes was significantly reduced after culture with the stimulation media compared to the control unstimulated media, while recombinant cytokines (TNFα + IFNγ, or IL-1β) had no significant effect (Supplemental Fig. 6).

## Discussion

The importance of inflammatory processes within the acute and chronic phase of tendinopathy has gained attention recently. However, a deeper understanding of the disease mechanisms including communication between tenocytes and immune cells is necessary. The current study shows that tenocytes isolated from human supraspinatus tendons change the expression level of surface markers and their cytokine release profile in response to complex stimulation mixtures originating from activated human immune cells as well as the single pro-inflammatory cytokines TNFα, IFNγ and IL-1β. Moreover, it was demonstrated for the first time that tenocyte communication with macrophages results in a varied surface marker expression pattern and modifies cytokine release levels. Our data are robust because we have a relatively large donor number of 10 compared to other studies, and we have controlled for the tenocyte source, sex and age of the donors, using only supraspinatus tendons from 46–69 (median 59.5) year old male patients, since age and sex have been shown to impact tenocyte biology^26, 27^.

Because previous studies detected pro-inflammatory cytokines such as IL-6, IL-8, IL-10, TNFα, and IL-17 in ruptured tendon samples^13, 28^, we first investigated the synthesis and impact of soluble pro-inflammatory cytokines on tenocytes. Tenocytes already constitutively produce IL-6 and MCP-1, but not IL-8, as previously described by other groups at the gene expression level^9, 29^. Stimulation with the single pro-inflammatory cytokines IL-1β, IFNγ or TNFα triggered the release of IL-8 and MCP-1, or sometimes both (Fig. 3c). In contrast, the stimulation of tenocytes with the complex cytokine mixture derived from αCD3αCD28 stimulated PBMCs led to a significant induction exclusively for IL-6 release (Fig. 2a).

Besides the secretion of soluble factors, we could show that after stimulation of the tenocytes *in vitro* with either a stimulation media derived from PBMCs or single pro-inflammatory cytokines, there were enhanced expression levels of adhesion molecules like ICAM-1 and VCAM-1, as well as the HLA-molecules HLA-ABC and HLA-DR (Figs. 1e and 3a). These results indicate changes in their cellular activation status thereby potentiating recruitment and interaction with other infiltrating cells during the inflammation process. Similar results have been found in studies with mesenchymal stromal cells^30^ and fibroblasts^31^, which share some of the same properties as tenocytes. In addition, our findings also agree with previous work showing that inflammation causes tenocytes to exhibit a pro-inflammatory phenotype which may lead to the development of chronic inflammation^5^.

Pro-inflammatory cues could be originating from myeloid cells or other immune cell subsets (mast cells, T cells) that were shown to infiltrate into diseased tendon^5, 13^. Because macrophages in particular have been found associated with injured tendon tissue^7, 10, 11, 13^, we tried to mimic this situation by using co-cultures of tenocytes with macrophages to examine how their interplay affects surface markers of each cell type after contact, and measured the cytokines released. We therefore co-cultured macrophages with the tenocytes either directly or without cell contact in a transwell system and investigated the effects on both cell types. Pre-stimulation of the tenocytes before co-culture induced up-regulation of the typical M1 marker CD80 while down-regulating another M1 marker, HLA-DR (Fig. 5b). The M2-type marker CD206 was expressed, but not altered under the same conditions. Therefore, we speculate that factors elevated in tenocyte/macrophage co-cultures trigger a partial macrophage activation as shown by elevated CD80 expression, but the fact that HLA-DR expression is down-regulated instead implies that M1 polarization is incomplete. Macrophage polarization is a dynamic process and the status represents a continuum rather than a strict classification as the changing tissue microenvironment conditions can redirect and shape the polarization^32–34^. The HLA-DR level was only significantly reduced when tenocytes were pre-stimulated, implying that the tenocytes are further triggered by an inflammatory environment to make soluble factors which regulate the behavior of the neighbouring macrophages. The soluble factor(s) involved have yet to be identified, but our findings indicate that tenocytes influence whether macrophages are directed towards more of a mixed M1/M2 phenotype following their initial activation during inflammation. In a model of equine tendon repair, a phenotype switch towards M2-type macrophage polarization along with reduced expression for the Lipoxin A4 receptor was shown recently for chronic injury and was described as indicative of incomplete inflammation resolution^21^.

Interestingly, tenocytes also showed significantly increased amounts of the adhesion molecules ICAM-1 and VCAM-1 after co-culture with macrophages (Supplemental Fig. 5). In parallel with altered surface markers, the supernatants of the macrophage/tenocyte direct co-cultures showed elevated levels of IL-6, IL-8, and MCP-1, as was detected after direct stimulation with pro-inflammatory cytokines. This likely originates mainly from the tenocytes since M0-type macrophages alone showed no IL-6 release and very low levels of IL-8 and MCP-1 (Fig. 4c). Notably, co-cultures did not show the typical M1-type cytokine profile with high levels of TNFα as indicated in other studies^22, 35, 36^. In addition, low levels of TGF-β1 were detected, with a trend towards higher levels in tenocyte/macrophage co-cultures (data not shown). TGF-β isoforms have been reported to play a crucial role during tendon development^37, 38^ and TGF-β1 could be an inducer of tenogenic regeneration^39^. M2 macrophages are known to secrete much less of the pro-inflammatory cytokine TNFα than M1 macrophages^36^, and instead produce various combinations of IL-10, TGF-β, IL-4, and IL-6 depending on the polarization agents used *in vitro* ^32, 34, 35^. Therefore, even though CD80 is elevated, the lack of TNFα along with the down-regulation of HLA-DR especially under inflammatory conditions fits with more of a mixed M1/M2 phenotype.

Another cytokine that may play an important role in the communication between tenocytes and macrophages is IL-6, as a significant increase in IL-6 release was measured when tenocytes were cultured with pro-inflammatory media from αCD3αCD28 stimulated PBMCs (Fig. 2a) and in the macrophage co-cultures (Fig. 4c). Previously, IL-6 has been implicated in the wound healing process, and tendon healing was reduced in IL-6 knock out mice, which showed impaired mechanical properties^13, 29^. This small glycoprotein exhibits some special immunological features having both pro- and anti-inflammatory properties depending on the type of signaling induced, either by classical binding or a form of trans-signaling via soluble IL-6 receptor (sIL-6R)^40–43^. Direct stimulation of tenocytes with recombinant IL-6 alone did not alter the surface marker expression (Supplemental Fig. 3) as was observed using the pro-inflammatory media from αCD3αCD28 stimulated PBMCs (Fig. 1). We therefore hypothesize that the IL-6 released by tenocytes is unable to trigger the tenocytes directly via classical signaling, but that they can acquire responsiveness to IL-6 when sIL-6R is present. In this way, the sIL-6R released from the immune cells could adopt the role of an “alarmin” as suggested by others^44^. Since granulocytes, macrophages, and other immune cells are present in the degenerated tendon^7, 8, 10^, this scenario is quite possible. This hypothesis is supported by the fact that another study described increased IL-6 mRNA expression, but reduced levels of IL-6 receptor in ruptured Achilles tendon and painful tibial tendon compared to normal tendon^45^.

Moreover, the level of IL-6 could also have an impact on tenocyte collagen I synthesis^46^. Rabbit tenocytes had elevated collagen I and III synthesis when combined with soluble factor mixtures or platelet rich plasma containing leukocytes^47^, supporting the role of interaction with immune cells and their released factors for affecting tenocytes under pathological conditions. We found an elevated release of IL-6 in tenocyte/macrophage co-cultures when tenocytes were pre-stimulated (Fig. 4c), and the collagen I level increased significantly during direct co-culture (Fig. 6c). This indicates that IL-6 could be supporting the ability of the tenocytes to produce collagen even during co-culture. In contrast to the co-culture setting, a significantly reduced collagen I production was detected after the treatment of tenocytes alone with the stimulation media (Supplemental Fig. 6). There was also a trend towards less collagen with single recombinant pro-inflammatory cytokines, underscoring the role of direct contact between tenocytes and macrophages in collagen production. Persistence of inflammatory macrophages would cause sustained and excessive matrix production and tissue fibrosis and may only exacerbate the tendon disorder^32, 47^. Therefore, the detection of elevated IL-6 levels combined with the mixed M1/M2 phenotype of the macrophages observed in our co-cultures might reflect part of a possible mechanism responsible for the development of chronic inflammation in tendons.

**Figure 7:**
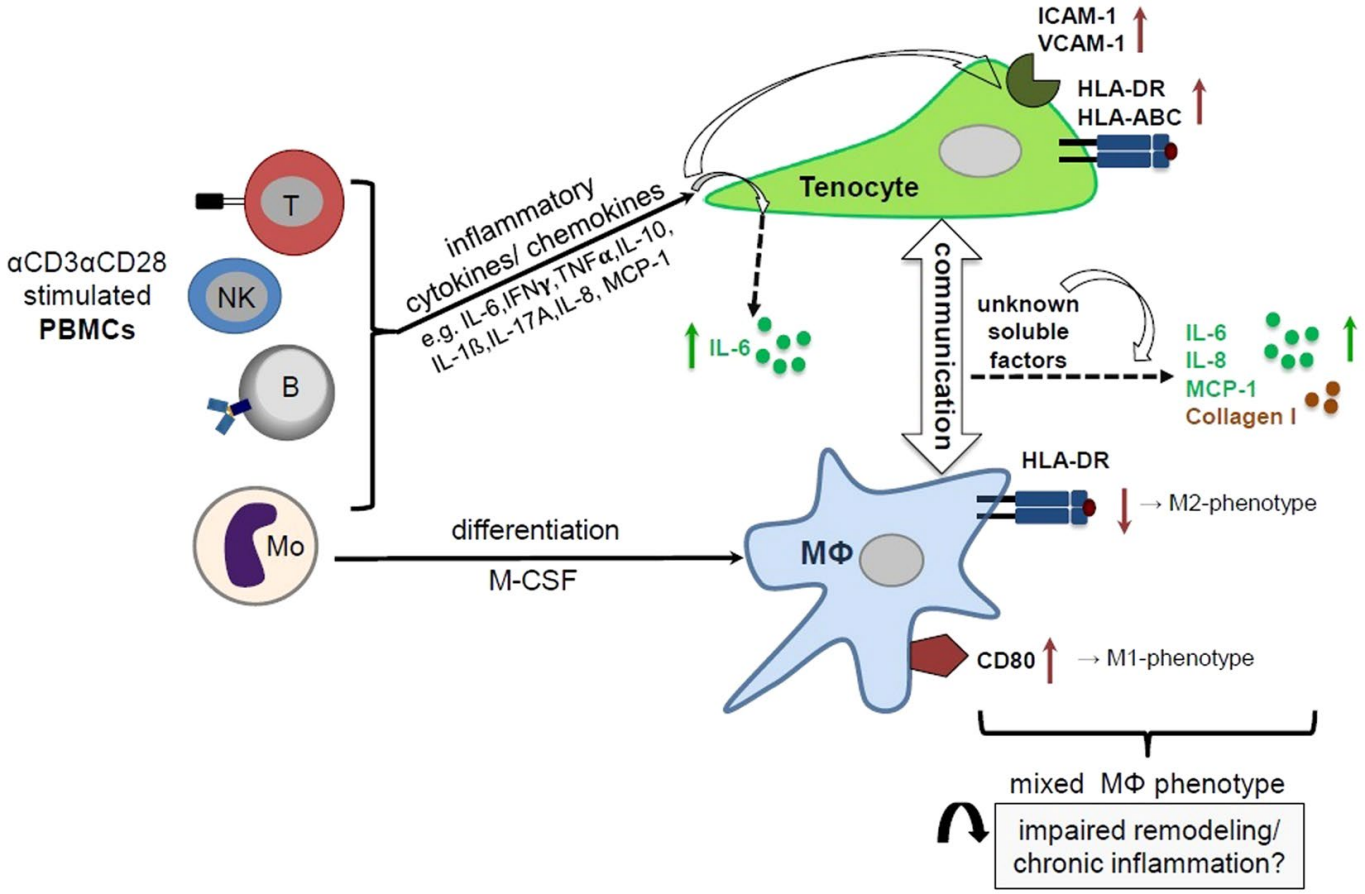
Potential role of cross-talk between tenocytes and macrophages in an inflammatory milieu in tendinopathy. The scheme illustrates the effects of inflammatory conditions on human tenocytes alone and during their interplay with human macrophages on surface marker expression and cytokine release patterns. A mixed cytokine stimulation originating from αCD3αCD28 activated human PBMCs including all subsets (T cells, B cells, NK cells and monocytes (Mo)) induces the up-regulation of HLA-molecules (HLA-DR, HLA-ABC) and adhesion molecules (ICAM-1, VCAM-1) on tenocytes and enhances their IL-6 secretion. After co-culturing monocyte-derived macrophages (Mϕ) with pre-stimulated tenocytes, the macrophages display a mixed M1/M2 macrophage phenotype with enhanced expression of CD80 (characteristic for M1-Mϕ), but reduced HLA-DR (characteristic for M2-Mϕ). As yet unknown soluble factors lead to higher levels of IL-6, IL-8 and MCP-1 secretion. We hypothesize, that this interaction might contribute to a disturbed resolution of the immune response after tendon injury leading to chronic tendinopathies.

The current study shows several changes in the cytokine release pattern and macrophage surface marker expression after direct contact with tenocytes in co-cultures. Interestingly, the effects found were also present without direct cell contact (Fig. 6). This indicates that most effects are likely mediated by soluble factors and that direct contact is not needed for increasing CD80 and reducing HLA-DR on macrophages or for the increase in IL-6, IL-8 and MCP-1 release (Fig. 7). The interplay between tenocytes and macrophages observed appears similar to the recently described cross-regulation of synovial fibroblasts and macrophages under pro-inflammatory stimulation by TNFα^48^. In this scenario, undefined soluble factors from synovial fibroblasts were able to suppress the IFN responses and signaling pathways of macrophages, which had an impact on their survival and polarization.

The current study was limited to tenocytes isolated from patients undergoing tendon tear repair. An equal number of donors with high versus low Goutallier degeneration scores were included. Though no differences were found in any *in vitro* tests for tenocytes with lower degeneration scores (data not shown), further studies are required to determine whether tenocytes isolated from healthy areas of tendon would respond similarly in an inflammatory environment. Additional experiments could also help to better elucidate the mechanism of the described changes in surface marker expression and identify the soluble factor(s) responsible. Deeper understanding of the signaling pathways involved would help to explain dysregulated tissue repair in inflammatory settings.

## Conclusion

We have demonstrated that human tenocytes do respond to pro-inflammatory cytokine stimulation by varying their expression of surface markers, and by secreting cytokines themselves. In addition, we found that as yet unknown soluble factors regulate the communication between tenocytes and macrophages in an inflammatory environment affecting the surface marker pattern of both cell types, as well as cytokine and collagen production. Understanding these interactions and identifying the soluble factors involved in future experiments could be beneficial in examining the mechanisms that retard the resolution of the immune response after tendon injury leading to chronic tendinopathies.

## Methods

### PBMC isolation

Human peripheral blood mononuclear cells (PBMCs) were isolated from buffy coats using a Biocoll density gradient (Biochrom, Berlin, Germany) with centrifugation at 800 g for 30 minutes at room temperature with no brake. Buffy coats from healthy volunteers were obtained with written informed consent by the German Red Cross Organization, Berlin, Germany. The use of buffy coats in the study was approved by the local ethics committee of the Charité-Universitätsmedizin Berlin as EA 1/226/14. After PBMCs were harvested from the interphase and washed three times with cold phosphate-buffered saline (PBS; Biochrom), they were either used for CD14^+^ monocyte isolation or to prepare stimulated supernatants.

### Generation of a stimulation media

Freshly isolated PBMCs were seeded with 1 million cells per well into a 24 well plate (Corning® Life Sciences B.V, Amsterdam, The Netherlands) containing 2 mL of PBMC media made from very low endotoxin (VLE) Roswell Park Memorial Institute (RPMI; Biochrom) with 10% human male heat-inactivated AB serum (Sigma-Aldrich, St. Louis, Missouri, U.S.A.), 100 U/mL penicillin, 100 μg/mL streptomycin, and 1% glutamine (Gibco®, Life technology™, Thermo Fisher Scientific; Waltham, Massachusetts, USA). The PBMCs were stimulated with 0.5 μg/mL anti-human CD3 (Janssen-Cilag, Neuss, Germany) and 1 µg/mL anti-human CD28 (BD Biosciences, Heidelberg, Germany) or left unstimulated as a control, and incubated at 37 °C with 5% CO_2_ for three days.

Supernatants from either αCD3αCD28 stimulated PBMCs or unstimulated controls were removed after the 3 day incubation, centrifuged at 300 g for 10 minutes at 4 °C to remove any remaining cells and cell debris, aliquoted and frozen at −80 °C. The comparison of proliferation responses in either unstimulated or αCD3αCD28 stimulated PBMC cultures and the composition of cytokines released from αCD3αCD28 stimulated PBMC cultures is shown in Supplemental Fig. 1. The unstimulated PBMC cultures contained around 50 pg/mL of IL-8 and MCP-1, and lacked the other cytokines (data not shown). Freshly thawed aliquots from the same batch of unstimulated or αCD3αCD28 stimulated supernatant media were used for each experiment.

### Tenocyte cultures

Supraspinatus samples from 10 male patients 46–69 (median 59.5) years old undergoing surgical debridement and repair of a tendon tear were collected after obtaining their written informed consent. The Bateman score for the tear size ranged from 2–4 for the ten patients (2: n = 4, 3: n = 2, 4: n = 4). Five patients had low (0: n = 4, 1: n = 1) and five patients had high (3: n = 2, 4: n = 3) scores for Goutallier fatty degradation. About half of the patients received treatment with either nonsteroidal anti-inflammatory drugs (NSAIDs) or Cortisone, or both at least once before surgery. Tissue harvest was performed according to the local guidelines of the Charité-Universitätsmedizin Berlin and the study protocol was approved by the ethics committee of the Charité-Universitätsmedizin Berlin (EA4/035/14). Tenocytes were isolated as described previously^49^ and cryopreserved. Cells were routinely passaged once before performing assays and used between passages 3–4. Tenocytes were cultured in ‘tenocytes media’ consisting of 1:1 DMEM/HamsF12 without glutamine (Biochrom) containing 10% heat-inactivated fetal calf serum (FCS; Hyclone Laboratories, GE Healthcare Life Sciences, Logan, Utah, USA), 100 U/mL penicillin, 100 μg/mL streptomycin, and 1% L-glutamine (Gibco®, Life technology™, Thermo Fisher Scientific) at 2.5 × 10^4^ cells/well in a 24 well plate in 1 mL tenocytes media. After the tenocytes were allowed to adhere overnight, they were stimulated with 1 mL of the control (unstim. media) or αCD3αCD28 stimulated supernatant (stim. media). Further wells of seeded tenocytes were stimulated by the addition of cytokines at a final concentration of 10 ng/mL in 2 mL tenocytes media either for IFNγ, TNFα, IFNγ and TNFα in combination, or IL-1ß (all cytokines from Miltenyi Biotec GmbH, Bergisch Gladbach, Germany).

After 3 days, supernatants were collected and frozen at −80 °C until use in cytokine detection assays and photos were taken with an AxioObserver microscope running AxioVision software (both from Carl Zeiss Microscopy GmbH, Jena, Germany) of the cell cultures before FACS staining and measurement as described below.

### Macrophage generation

PBMCs isolated from buffy coats were resuspended in 1 mL of ice-cold MACS buffer (PBS containing 1% FCS and 2 mM ethylenediaminetetraacetic acid (EDTA; Sigma-Aldrich), vortexed, and conjugated with magnetic beads specific for human CD14 (Miltenyi Biotec). The cell suspension was incubated for 15 minutes at 4 °C and washed with media. Subsequently, the cells were filtered by a 30 µm pre-separation filter, applied to an LS-column (both Miltenyi Biotec) and placed within the provided magnet. The positive fraction was collected and a portion was stained with a human-specific CD14 PerCPCy5.5 antibody (Biolegend) to check the purity, which ranged between 93–97%, while the rest were cryopreserved. These frozen monocytes were thawed, washed three times with cold PBS and then seeded as 1 million cells per well of a 6 well plate (Corning) in PBMC media (VLE RPMI containing 10% AB serum, 100 U/mL penicillin, 100 μg/mL streptomycin, and 1% glutamine) with 50 ng/mL macrophage colony-stimulating factor (M-CSF; Miltenyi Biotec) and incubated for 6 days to become M0-type macrophages.

### Tenocyte/macrophage co-cultures

Six well plates were seeded with 0.125 million tenocytes one day before 0.625 million M0-type macrophages were added for a ratio of 1 tenocyte:5 macrophages and co-cultured for 3 days in a 50:50 mix of tenocytes media and PBMC media. The ratio of 1:5 was chosen since after 3 days of culture, the number of tenocytes and macrophages would be similar after the tenocytes proliferate in culture, while the macrophages do not proliferate. Additionally, some tenocytes were pre-stimulated for 3 days with a 50:50 media mixture of tenocytes media and the αCD3αCD28 stimulated supernatant before initial seeding into 6 well plates, and were designated as pre-stimulated tenocytes (Fig. 4a). Control wells containing the same number of M0-type macrophages were seeded alone in a 50:50 mix of tenocytes media and PBMC media. After 3 days, photos were taken and supernatants were collected and frozen at −80 °C until use in cytokine detection assays. Cells were harvested and stained for further FACS analysis as described below. To determine whether effects were contact dependent, some assays were repeated using tenocytes in a transwell system in parallel with direct co-cultures. Tenocytes were seeded into 0.4 µm pore size inserts (Corning) and co-cultured with M0 macrophages as described above.

### Fluorescence staining of cells and flow cytometry measurement

Non-adherent PBMCs were resuspended by pipetting, and adherent cells were harvested using either a 0.05% trypsin solution with EDTA or Accutase (both Gibco®, Life technology™, Thermo Fisher Scientific) and transferred to 5 mL FACS tubes (Falcon). Briefly, cells were washed once with cold PBS and resuspended in a final volume of 50 µL antibody mix in cold FACS buffer (PBS supplemented with 1% FCS) for 30 minutes at 4 °C in the dark. Antibody panel I consisted of ICAM-1 (CD54) FITC, VCAM-1 (CD106) PE, CD45 Pacific Blue, HLA-DR PECy7, CD90 APC, and HLA-ABC PerCP. Panel II contained CD105 FITC, CD29 PE, CD90 PerCPCy5.5, CD44 PECy7, and CD73 APC. When macrophages were added, panel III was used which contained CD90 FITC to identify tenocytes, as well as the macrophage markers CD14 APCCy7 and CD16 PerCPCy5.5, the M1 macrophage polarization markers CD80 PE and HLA-DR PECy7, and the M2 polarization marker CD206 APC. All antibodies were purchased from Biolegend, and dilutions for antibody panels I, II and III are given in Supplemental Table 1. Antibody mixes also contained either the Live/Dead^®^ violet or aqua Fixable Staining Kits (Molecular probes™, Thermo Fisher Scientific) in order to exclude dead cells from the analysis. After incubation, the samples were washed with cold FACS buffer and resuspended in 1% paraformaldehyde (PFA; Roth, Karlsruhe, Germany) in FACS buffer. Samples were kept at 4 °C in the dark until measurement. Data collection was done using a FACS Canto II device with FACS Diva software (Becton Dickinson, San Jose, CA, USA). Data analysis was performed using FlowJo software (TreeStar Inc., Ashland, OR, USA). Gating strategies for the FACS-analysis of tenocytes and macrophages are shown in Supplemental Fig. 4.

### Cytokine and collagen detection assays

The supernatants were tested for cytokines using the Legendplex^TM^ human inflammation 13-plex panel (Biolegend) for IL-1β, IFNα, IFNγ, TNFα, MCP-1, IL-6, IL-8, IL-10, IL-12p70, IL-17A, IL-18, IL-23, and IL-33. The minimum detectable concentration of each cytokine is given as 0.6–2.1 pg/mL by the manufacturer, but values less than 10 pg/mL were considered irrelevant. Samples were treated following the manufacturer’s instructions and measured with a FACS Canto II device (BD Biosciences). Analysis was done using Legendplex software (Biolegend). Supernatants were tested for type I Collagen using the MicroVue CICP EIA Kit for determining levels of CICP (C-Terminal of Type I Collagen; Quidel, San Diego, CA, USA) and CICP values were interpolated from the standard curve after measuring the optical density at 405 nm on a microplate reader (Bio-Rad, Munich, Germany).

### Statistics

Results are expressed as the median with interquartile range. Statistical analyses were chosen that do not rely on normal distribution because all data sets were n ≤ 10. Statistical differences were determined either using Mann-Whitney nonparametric *t-*tests between two groups with only one variable, or with the Kruskal Wallis non-parametric ANOVA with Dunn’s post-test for multiple variables, with P ≤ 0.05 considered statistically significant. Statistical analysis was performed using GraphPad Prism 6.0 software (GraphPad Software, San Diego, CA, USA).

### Data Availability

MSt and MSe have access to all the data, and data are available upon request.

## Electronic supplementary material

Supplemental Information
