# Supplementary material for: New insights into tenocyte-immune cell interplay in an in vitro model of inflammation
Source: Sci Rep. 2017 Aug 29;7:9801. doi: 10.1038/s41598-017-09875-x (PMC5575127; doi:10.1038/s41598-017-09875-x)
Supplement: Supplementary file 1 — Supplemental Information [file 41598_2017_9875_MOESM1_ESM.pdf]

# Supplemental Information

# New insights into tenocyte-immune cell interplay in an *in vitro* model of inflammation

Meaghan Stolk[1], Franka Klatte-Schulz[1,2], Aysha Schmock[2], Susann Minkwitz[2], Britt Wildemann[1,2,+], Martina Seifert[1,3,*,+]

[1] Berlin-Brandenburg Center for Regenerative Therapies (BCRT), Charité-Universitätsmedizin Berlin, corporate member of Freie Universität Berlin, Humboldt-Universität zu Berlin, and Berlin Institute of Health, Berlin, 13353, Germany

[2] Julius Wolff Institute, Charité-Universitätsmedizin Berlin, corporate member of Freie Universität Berlin, Humboldt-Universität zu Berlin, and Berlin Institute of Health, Berlin, 13353, Germany

[3] Institute of Medical Immunology, Charité-Universitätsmedizin Berlin, corporate member of Freie Universität Berlin, Humboldt-Universität zu Berlin, and Berlin Institute of Health, Berlin, 13353, Germany

[*]martina.seifert@charite.de

[+]these authors contributed equally to this work

**Evaluation of the αCD3αCD28 stimulation media**

In parallel with the 3 day cultures used to generate the unstimulated and αCD3αCD28 stimulated supernatants, an additional 10 million PBMCs were labeled with 2.5 µM carboxyfluorescein succinimidyl ester (CFSE; Molecular probes™, Thermo Fisher Scientific; Waltham, Massachusetts, U.S.A.) for three minutes at room temperature. The staining reaction was stopped by adding cold FCS and subsequent incubation for one minute. After washing the cells twice with cold PBS, 3 x $10^5$ CFSE-stained PBMCs were seeded in a 24 well plate and either left unstimulated or stimulated with anti-CD3 and anti-CD28 as described above. After 3 days at 37°C and 5% $CO_2$, CFSE PBMCs were harvested and stained with anti-CD3 APCCy7 antibody (Biolegend, San Diego, California, U.S.A.) and subjected to flow cytometry (fluorescence activated cell sorting, FACS) to confirm the activation of T cell proliferation by the CD3CD28 stimulation (**Supplemental Fig. 1a**). The background proliferation of the unstimulated PBMCs was 2.7% of the viable cells, while the CD3CD28 stimulated cells had 60% proliferation. Both cultures consisted of approximately 75% CD3+ (T) cells. The cytokine content was tested using the Legendplex™ Human Inflammation panel, and the CD3CD28 stimulated supernatant, used as stimulation media, showed detectible amounts of IL-6, IL-8, MCP-1, IFNγ, TNFα, IL-10, and IL-1β (**Supplemental Fig. 1b**), see Cytokine Detection Assays for details.

## Supplemental Figure 1

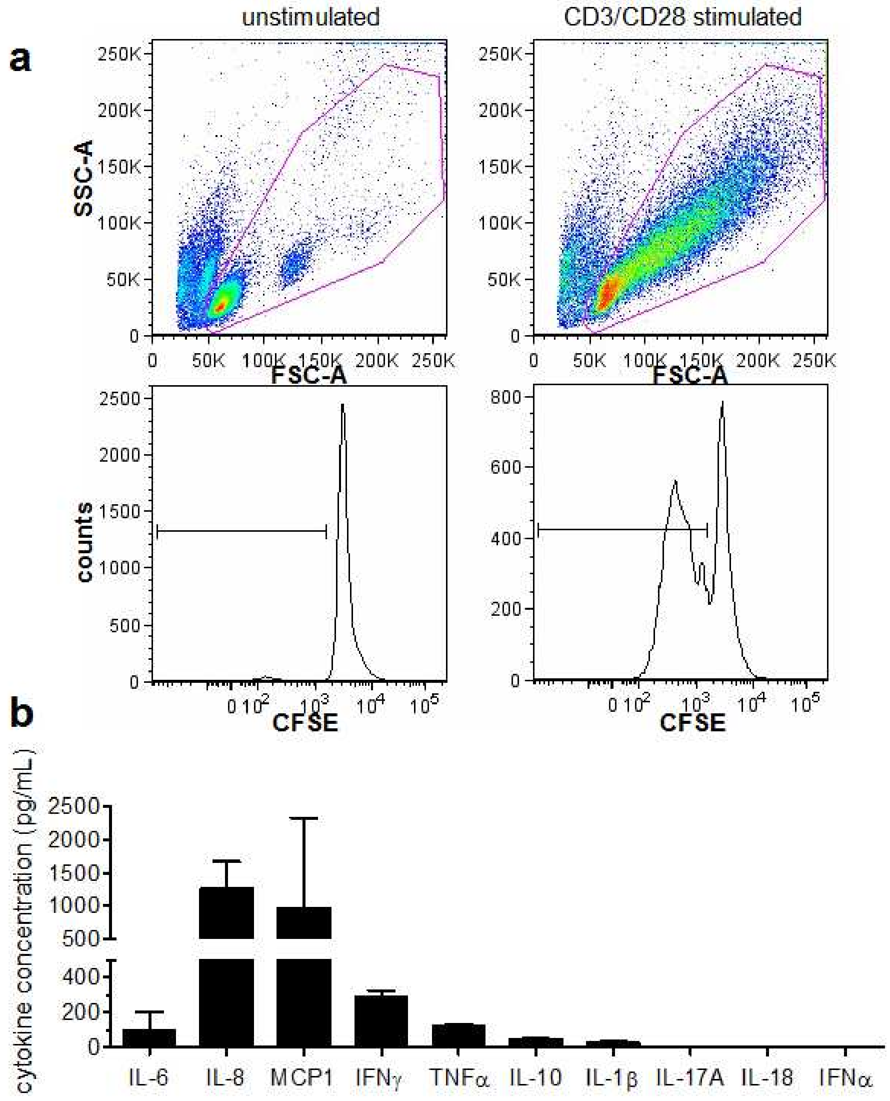


**Supplemental Figure 1. Proliferation of the αCD3αCD28 stimulated PBMCs and characterization of the stimulation media.**

Freshly isolated PBMCs from a buffy coat were stimulated with 0.5 µg/mL anti-human CD3 + 1 µg/mL anti-human CD28 in VLE-RPMI media containing 10 % heat-inactivated AB serum or left unstimulated as a control. The effectiveness of αCD3αCD28 stimulation on proliferation of CD3 APCCy7$^+$ T cells was confirmed using a CFSE-based assay and measured by flow cytometry **(a)**. Media from either unstimulated or αCD3αCD28 stimulated PBMCs was removed after 3 days incubation, aliquoted, and frozen at -80°C. Freshly thawed aliquots from the same batch of unstimulated (unstim. media) or αCD3αCD28 stimulated media (stim. media) were later used to stimulate tenocytes, and the cytokines in the αCD3αCD28 stimulated media are shown after measurement with the Legendplex™ human inflammation panel for IL-1β, IFNα, IFNγ, TNFα, MCP-1, IL-6, IL-8, IL-10, IL-12p70, IL-17A, IL-18, IL-23, and IL-33.

# Supplemental Figure 2

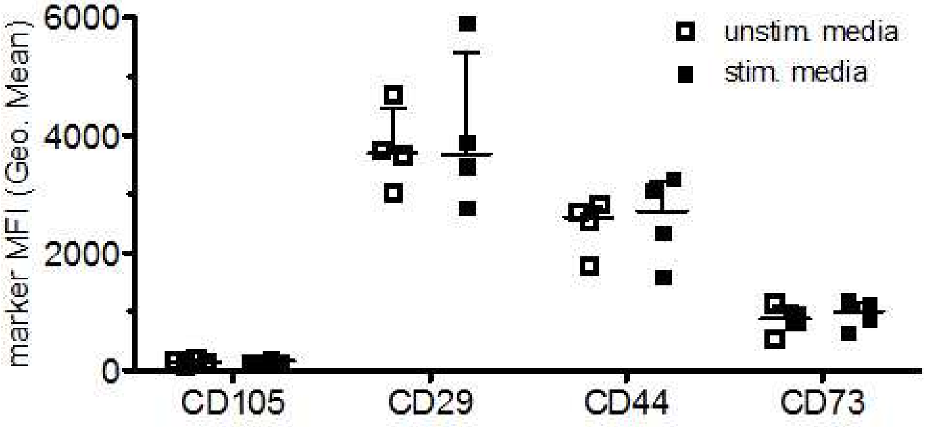


**Supplemental Figure 2. Stromal cell surface markers expressed on tenocytes are not affected by a pro-inflammatory stimulation media.**

Following incubation for 3 days with either the control media from unstimulated PBMC (unstim. media) or the pro-inflammatory stimulation media from αCD3αCD28 stimulated PBMCs (stim. media), the tenocytes were stained with a panel of human-specific antibodies to CD105, CD29, CD44, and CD73 and analyzed by flow cytometry. Data are presented as the median of n=10 with the interquartile range and are considered significantly different when *$p<0.05$ with the Mann-Whitney non-parametric t-test.

# Supplemental Figure 3

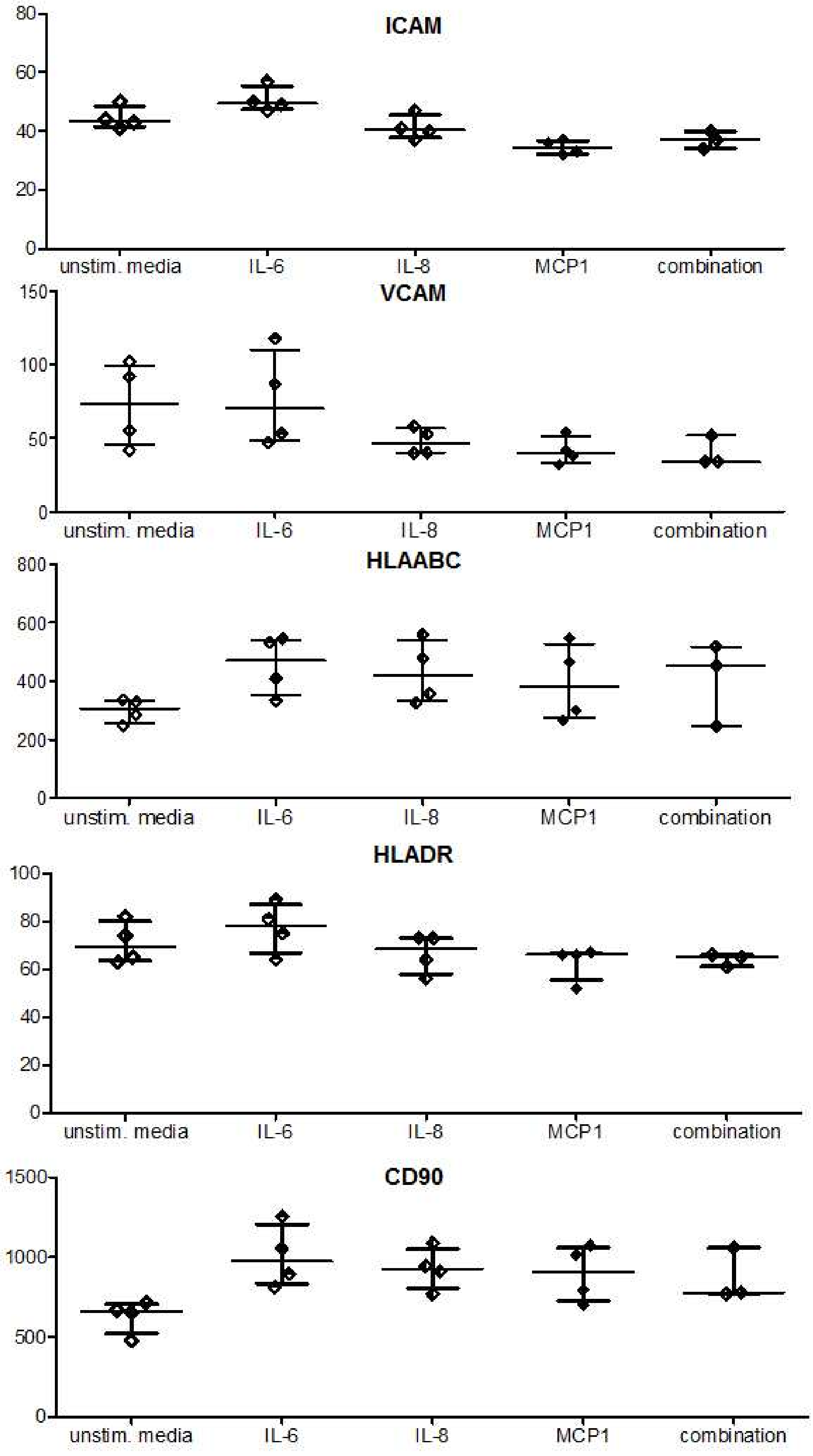

**Supplemental Figure 3. The predominant cytokines found in the pro-inflammatory stimulation media do not affect the surface marker expression of tenocytes.**

Following incubation for 3 days with either the unstimulated control media (unstim. media) or a 10ng/mL final concentration of the recombinant cytokines IL-6, IL-8 and MCP1 alone or in combination, the tenocytes were harvested and stained with a panel of human-specific antibodies to characteristic surface markers and analyzed by flow cytometry. The mean fluorescence intensity (MFI) of the adherent molecules ICAM-1 (CD54) and VCAM-1 (CD106), as well as the HLA class I molecule HLA-ABC, the HLA class II molecule HLA-DR, and CD90 (Thy-1) are shown. Data are presented as the median of n=10 with the interquartile range and are considered significantly different when *$p<0.05$ with the Kruskal Wallis non-parametric ANOVA with Dunn's post-test.

# Supplemental Figure 4

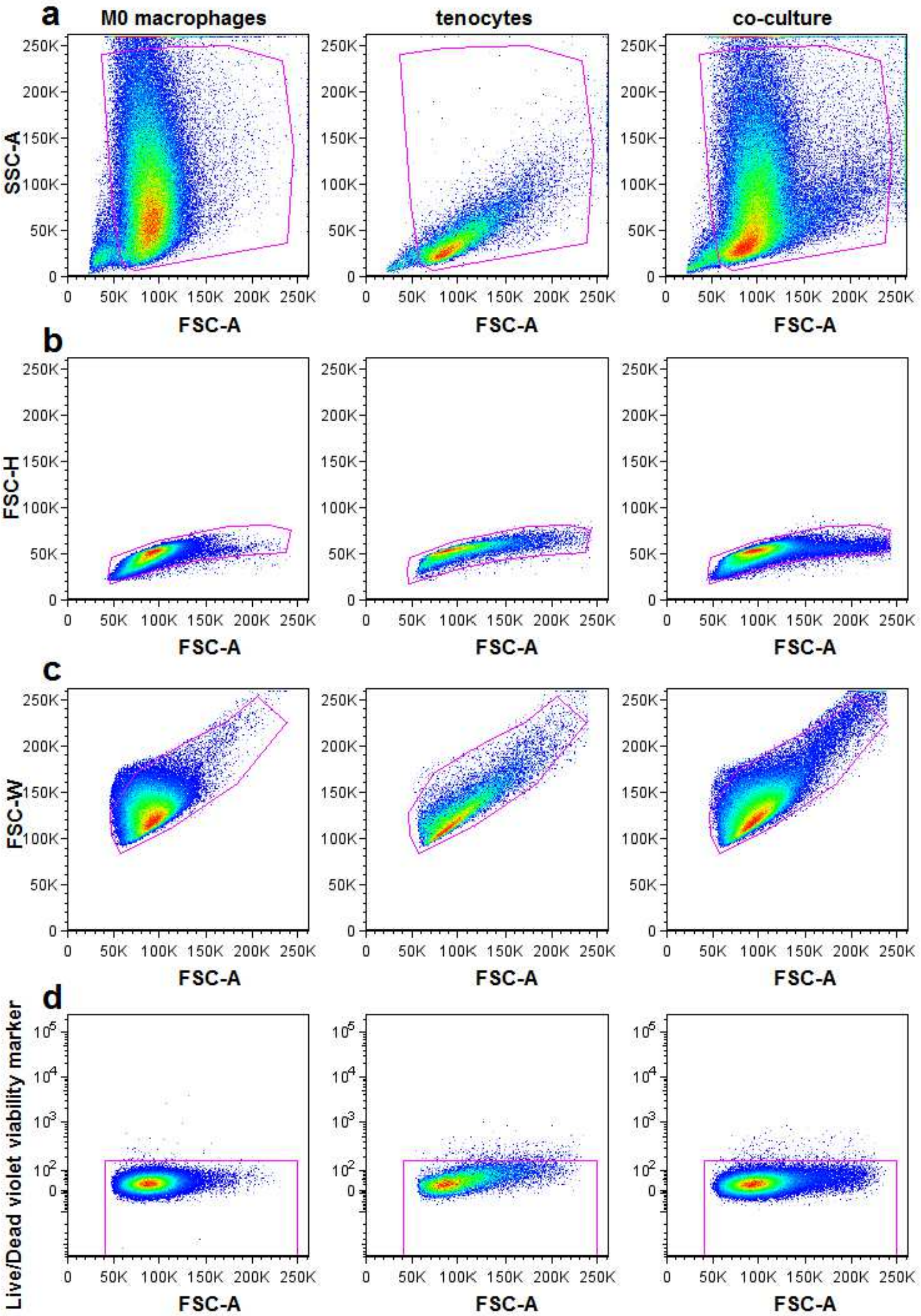


**Supplemental Figure 4. Representative plots illustrating the gating strategy for tenocyte/macrophage co-culture flow cytometry data.**

After excluding debris in the FSC-A vs SSC-A plot **(a)**, doublets were excluded by examining FSC-A vs FSC-H **(b)** and FSC-A vs FSC-W plots **(c)**, before further gating to exclude any dead cells using the Live/Dead violet viability marker **(d)**.

# Supplemental Figure 5

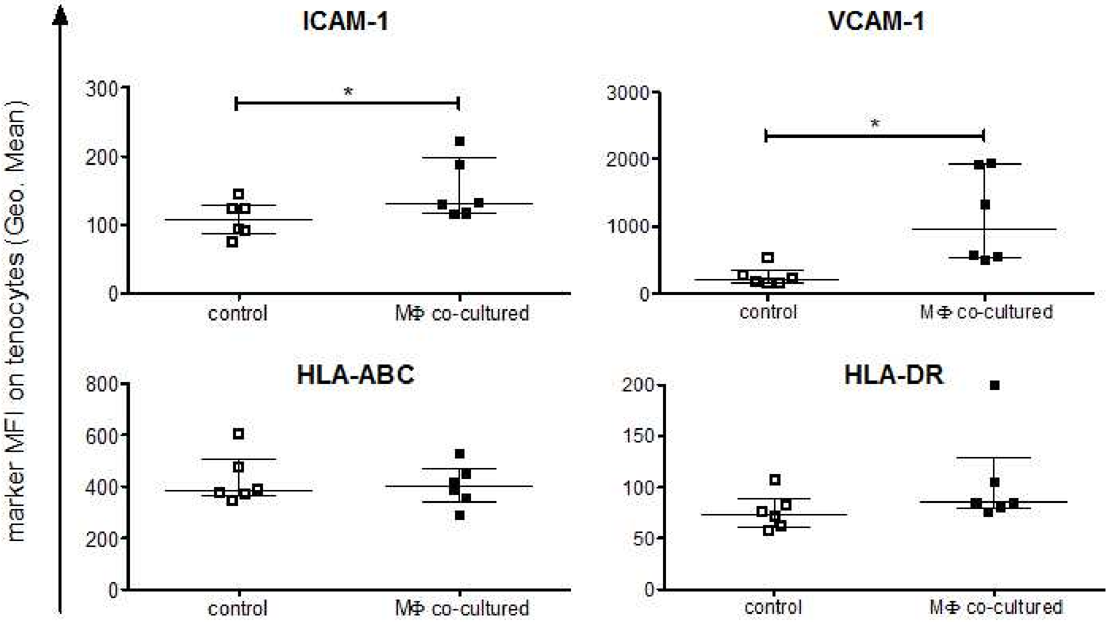


**Supplemental Figure 5. Co-culture with macrophages changes the surface marker expression on tenocytes.**

Tenocytes were seeded overnight in 6 well plates and then M0-type macrophages were added for a ratio of 1 tenocyte to 5 macrophages. After 3 days, cells were stained with a panel of human-specific antibodies to characteristic surface markers by flow cytometry. Tenocytes were identified from the co-cultures by gating on the $CD90^+$ population before evaluating the mean fluorescence intensity (MFI) of ICAM-1 (CD54), VCAM-1 (CD106), HLA-ABC, and HLA-DR on co-cultured tenocytes (MΦ co-cultured) in comparison to tenocytes that were cultured alone (control) under the same conditions.

Data are presented as the median with the interquartile range of n=6 and are considered significantly different when *$p<0.05$ with the Mann-Whitney non-parametric t-test.

## Supplemental Figure 6

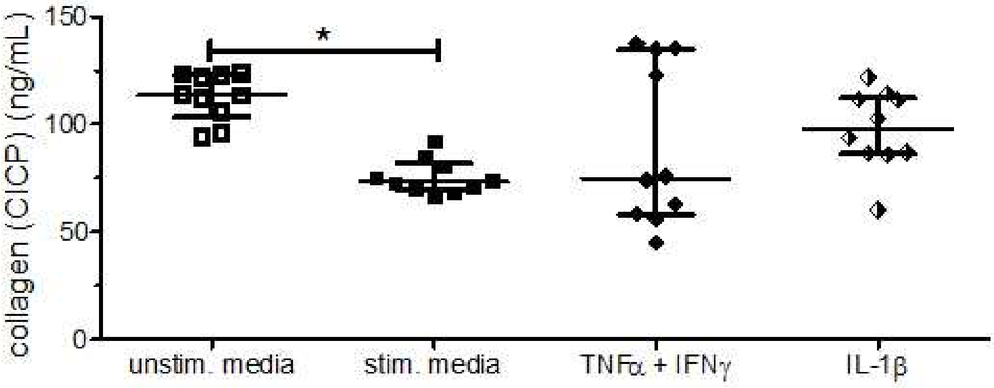


**Supplemental Figure 6. Stimulation media reduces collagen synthesis, but recombinant cytokines have no effect.**

Supernatants were tested for CICP (C-terminal of type I collagen) using an EIA Kit following the incubation of tenocytes for 3 days with either the control media from unstimulated PBMC (unstim. media), the pro-inflammatory stimulation media from αCD3αCD28 stimulated PBMCs (stim. media), recombinant TNFα + IFNγ used in combination, or IL-1β alone at final concentrations of 10 ng/mL. Data are presented as the median with interquartile range for n=10 and are considered significantly different when *$p<0.05$ with the Kruskal Wallis non-parametric ANOVA with Dunn's post-test.

**Supplemental Table 1: Antibody Panels Table**

| Antibody panel | Marker | Fluorochrome | Dilution |
|---|---|---|---|
| **Panel I** | ICAM-1 (CD54) | FITC | 1:1000 |
| | VCAM-1 (CD106) | PE | 1:50 |
| | CD45 | Pac Blue | 1:500 |
| | HLA-DR | PECy7 | 1:200 |
| | CD90 | APC | 1:600 |
| | HLA-ABC | PerCP | 1:200 |
| | Aqua Live/Dead | 510 | 1:100 |
| **Panel II** | CD105 | FITC | 1:50 |
| | CD29 | PE | 1:100 |
| | CD90 | PerCPCy5.5 | 1:400 |
| | CD44 | PECy7 | 1:3000 |
| | CD73 | APC | 1:1000 |
| | Aqua Live/Dead | 510 | 1:100 |
| **Panel III** | CD90 | FITC | 1:50 |
| | CD80 | PE | 1:20 |
| | CD16 | PerCPCy5.5 | 1:200 |
| | CD206 | APC | 1:100 |
| | HLA-DR | PECy7 | 1:600 |
| | CD14 | APCCy7 | 1:100 |
| | Violet Live/Dead | V450 | 1:5000 |
